# Supplementary material for: Distinct Effects of Chemical Toxicity and Radioactivity on Metabolic Heat of Cultured Cells Revealed by “Isotope-Editing”
Source: Microorganisms. 2023 Feb 25;11(3):584. doi: 10.3390/microorganisms11030584 (PMC10056173; doi:10.3390/microorganisms11030584)
Supplement: Supplementary file 1 [file microorganisms-11-00584-s001.zip › microorganisms-2166666-supplementary.pdf]

Supplementary Materials

Distinct Effects of Chemical Toxicity and Radioactivity on Metabolic Heat of Cultured Cells Revealed by "Isotope-Editing"

Jana Oertel [1], Susanne Sachs [1], Katrin Flemming [1], Muhammad Hassan Obeid [2] and Karim Fahmy [1,3,*]

[1] Helmholtz-Zentrum Dresden-Rossendorf, Bautzner Landstrasse 400, 01328 Dresden, Germany
[2] Protection and Safety Department, Atomic Energy Commission of Syria, Damascus P.O. Box 6091, Syria
[3] Cluster of Excellence Physics of Life, Technische Universität Dresden, 01062 Dresden, Germany
* Correspondence: k.fahmy@hzdr.de

I. Growth of *Lactococcus lactis* in the presence of Eu

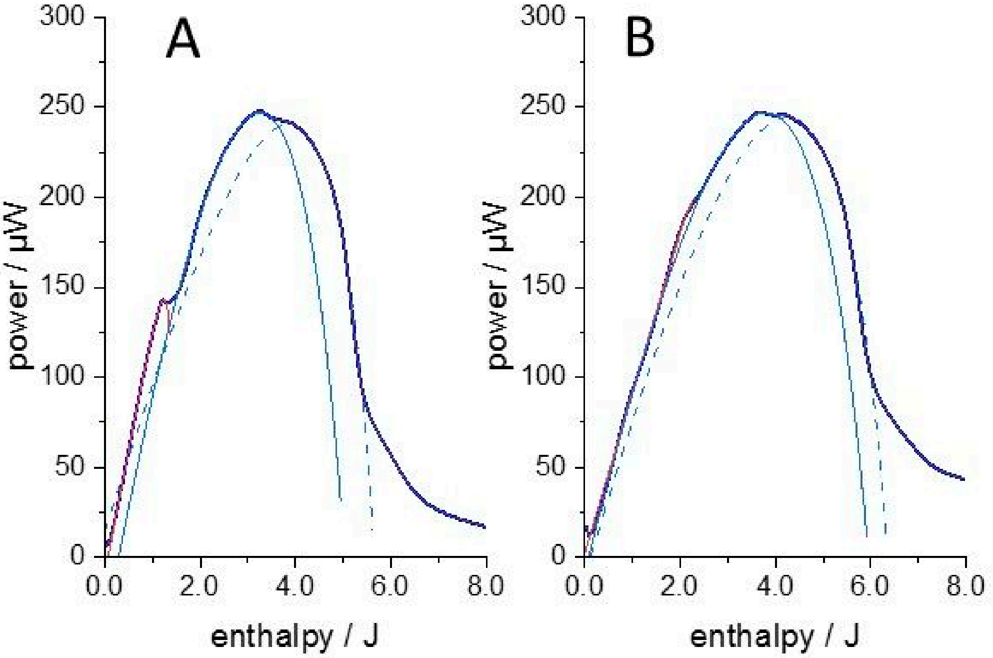

Figure S1: "Enthalpy plots" of the growth of *L. lactis* in the presence of ^{152}Eu. The medium contained 100 µM Eu in total and was supplemented with (A) 130 pM and (B) 630 pM ^{152}Eu. Data were recorded and analyzed as described in the main text (thin lines represent fits to three consecutive metabolic phases).

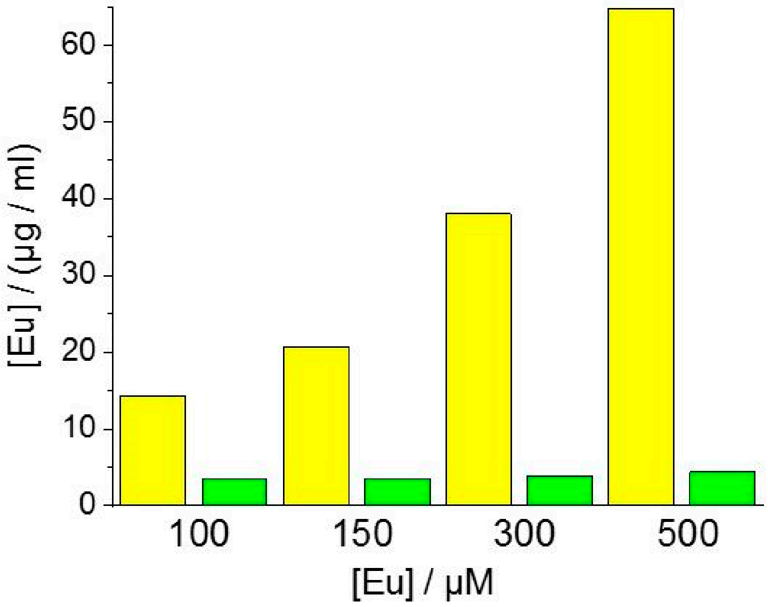

Figure S2: Europium concentration in *L. lactis* growth medium. Concentrations were determined by ICP-MS (see Materials and Methods in main text) using growth medium without (yellow) and with bacteria grown over 20 h (green) in the presence of several ^{153}Eu concentrations (0, 100, 150, 300 and 500 µM $EuCl_3$). The medium was centrifuged at 1000 x g for 10 minutes prior to ICP-MS to pellet insoluble complexes or bacteria before analysis of the supernatants.

II. Growth of *Brassica napus cells* in the presence of ^{233}U

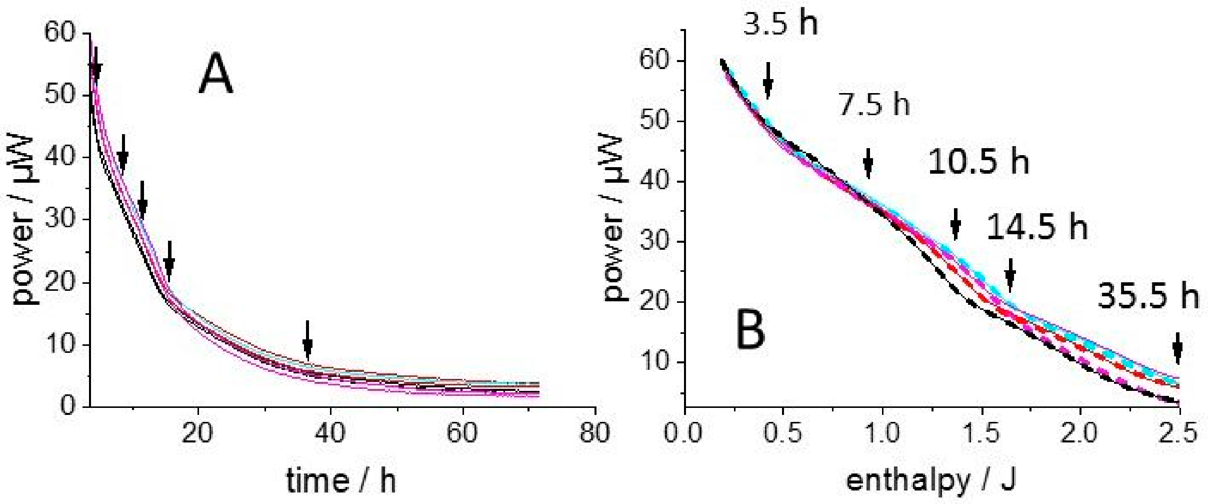

Figure S3: Thermograms of *B.* napus cells grown in the presence of uranyl(VI) nitrate. A) Time domain thermograms measured in the presence of 0 µM (black), 1 µM (red), 7 µM (cyan) and 15 µM (magenta) ^{233}U. The total uranyl(VI) concentration (adjusted by natural uranium) was of 50 µM for all samples. B) Enthalpy plots of the same data (dashed traces). Solid lines represent the enthalpy plot of the control (without ^{233}U) after rescaling it along the x and y-axis to best match the data recorded with ^{233}U. This procedure adjusts for small inevitable changes in cell quantity when weighing and transferring the previously washed cells to the calorimeter ampoules. The scaling shows that all thermograms exhibit the same general curvature with characteristic features at the indicated times. Only at the highest ^{233}U concentration of 15 µM, a clear deviation from the control was observed (magenta). The thermal power was reduced by the α-emitter below that of the control (dashed line lies below the solid line). The late occurrence of this deviation suggests that radio-damage accumulates over time rather than causing cell death for a certain fraction of cells already at the very beginning of the exposure to the radionuclide (as observed with bacteria, see Figure S5). Arrows indicate corresponding time points in the two plots.

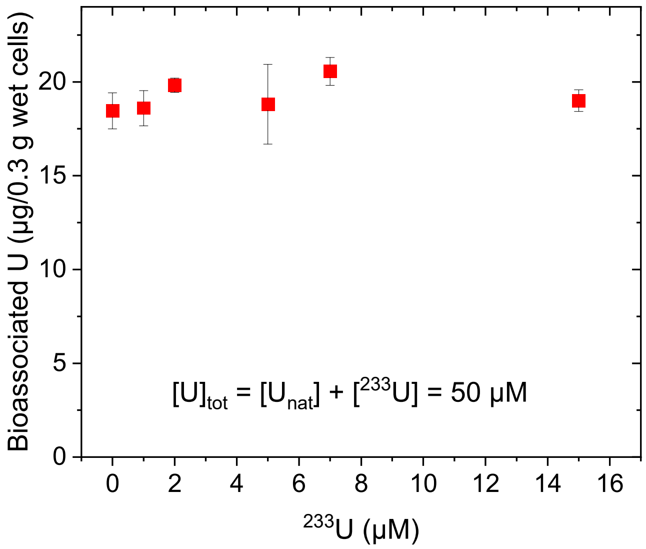

Figure S4: Estimated amount of cell-associated uranium after exposure of *B. napus* cells. The total concentration of uranium was kept at 50 µM. The concentration of ^{233}U was varied between 0 and 15 µM. Data represent mean values and standard errors of the mean.

III. Growth of *Lysinibacillus sphaericus* cells in the presence of ^{233}U

Lysinibacillus sphaericus cell culture.

Lysinibacillus sphaericus strain JG-B53 was cultured at 30 °C in 2 ml 50% R2A medium (containing, per liter, 0.25 g yeast extract, 0.25 g peptone, 0.25 g casamino acids, 0.25 g glucose, 0.25 g soluble starch, 0.15 g sodium pyruvate, 0.15 g K_2HPO_4, and 0.025 g $MgSO_4$ x $7H_2O$) with an initial OD600 of 0.0005 at the start of microcalorimetric measurements. Bacteria were exposed to different concentrations of natural uranium (Unat) in the form of $UO_2(NO_3)_2$ within liquid cultures. All samples were prepared in duplicates.

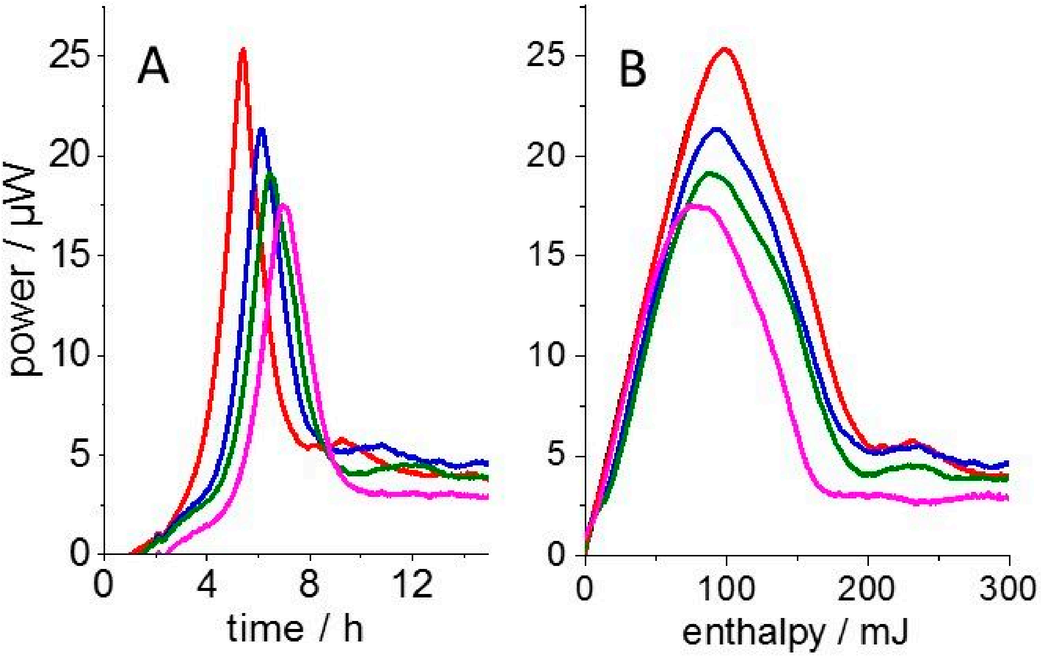

Figure S5: Thermograms of *Lysinibacillus sphaericus* strain JG-B53. A: Heat flow is plotted vs. time. Bacteria were cultured at 30 °C in 2 ml 50% R2A medium. ^{233}U was added as $UO2(NO_3)_2$ at 0 (red), 0.5 (blue), 1.0 (green) and 1.5 µM (magenta)final concentrations. B: "Enthalpy plot" showing the metabolic activity in dependence of released heat, i.e., a biomass- proportional scale. The curves correspond to the averages of duplicates. Natural uranium exhibited negligible influence on growth at these concentrations evidencing the detrimental effect of α-radiation on peak metabolic activity but not on the initial growth rate. The effect indicates that ^{233}U reduces the initial number of viable cells leading to corresponding time delays in (A) but allowed the population of initially unaffected cells to grow normally. Increased damage from α-particle radiation occurred only above a critical cell density (reached after a total heat release of 50 mJ to 100 mJ) probably due to an increasing number of cells occupying the volume in which they come into reach of radiation from ^{233}U bound to the surface of the initially killed population of bacteria. All traces were obtained from duplicate experiments.
